# Supplementary material for: Facilitators and barriers to linkage to HIV care and treatment among female sex workers in a community-based HIV prevention intervention in Tanzania: A qualitative study
Source: PLoS One. 2019 Nov 19;14(11):e0219032. doi: 10.1371/journal.pone.0219032 (PMC6863533; doi:10.1371/journal.pone.0219032)
Supplement: S1 Text — (DOC) [file pone.0219032.s001.doc]

# DATA COLLECTION TOOLS

**2.1. Focus Group Discussion Guide: FSW & vAGYWs**

**Understanding facilitators and barriers to partner notification, Linkage to Care and Treatment in the Sauti program implementation regions in Tanzania**

Participants’ demographics –Facilitator will fill out the following chart by speaking with each participant prior to initiating the focus group discussion

| **Number** | **Age** | **Level of education** | **Marital status** | **Main activity/sources of income** | **Remarks** |
| --- | --- | --- | --- | --- | --- |
| 1 |  |  |  |  |  |
| 2 |  |  |  |  |  |
| 3 |  |  |  |  |  |
| 4 |  |  |  |  |  |
| 5 |  |  |  |  |  |
| 6 |  |  |  |  |  |
| 7 |  |  |  |  |  |
| 8 |  |  |  |  |  |
| 9 |  |  |  |  |  |
| 10 |  |  |  |  |  |

**Instruction for the facilitators:** These questions are guides for the discussion. In general, please try to follow the question flow outlined below. Participants may take the conversation in a different direction. This is fine, but please make sure that by the end of the session you have covered all of the listed domains.

**Introductory text for group:** Thank you all for coming today and agreeing to speak with us. Everything we discuss today will be confidential so we ask you not to discuss what we talk about with anyone outside of this room. You have kindly agreed for us to make an audio recording of our discussion and in order to protect your privacy, we ask you not to mention your names or anyone else’s name during the session. Instead please use the number/letter that we just assigned to identify yourself when you want to speak.

| **Theme** | **Q** | **Questions** | **Summary** |
| --- | --- | --- | --- |
| **Understanding partner notification** |  | I would like to begin by discussing issues related to communication with partners.  What are important issues that girls/women discuss with their sexual partners?  Probe for:   - protecting oneself from getting pregnancy - testing for HIV - Using other means of protection – (to be mentioned)   How do partners react when a girl/woman initiates a discussion about those issues? Give examples.  How if partners would know each other’s HIV status? (*is it something good/bad?)  Probe for:   - What would happen if one is found HIV positive - What would happen if both are HIV positive or negative?   What are the possible ways that can be used to reach your partner for HIV testing? Why?  Probe for:   - Having biomedical providers contacting the partner without revealing her status and why? - Bringing the partner directly for testing and why? - Any assistance required for the partner to come and why? |  |
| **Context of HIV test & care seeking practice** |  | I want to understand a bit about your experience in making decisions to test for HIV and use of medicines in your community.  Think about a typical girl/woman in your community, what can influence her to go for a HIV test?  Probe for:   - How can she feel suppose she test HIV positive? Give examples - What can she do after the positive results?   What can one do to continue living after having tested HIV positive?  Probe for:   - Are there medicines that one can use for that condition? (Knowledge on availability of ART) - Where can one access care and treatment services - What are the challenges one can expect in accessing these services? Give examples - What would happen if one delays in seeking care? |  |
| **Care and treatment context** |  | Have you ever heard about care and treatment facilities? [if this was mentioned, re-phrase as:  You mentioned that sometimes girls/women go to care and treatment facilities......  What are their experiences in accessing the services there?  Probe for:   - How do they describe the quality of services offered? Give examples - How do they say about healthcare providers there? Pay attention to stigma and discrimination.   What expenses (monetary or non-monetary) would a girl/woman incur in attending and receiving care and treatment? |  |
| **Barriers and facilitators to linkage to care and treatment for people diagnosed with HIV** |  | I'd like to understand the context of linkage to care and treatment among girls/women. Let us think about a girl called Asha who lives in this village/town.  **Read the vignette aloud:**  ***“Asha is a young woman age 17. She lives with a man as couples and has no source of income apart from what she receives from the man. Asha accepted to take a HIV test provided by Sauti project, and was diagnosed HIV positive. After the test, the project staff told her that they will bring her to the health care facility located in their town/village for counselling and treatment initiation. However, Asha did not accept the offer. Asha received a sum of 5,000/- shillings so that she can go by herself. After six days the project staff made a follow-up to know how Asha was doing, but, they found that Asha did not go to the health facility for her HIV counselling and medication”.***  What may have prevented Asha from going to the health facility for initiation of care and treatment? Give examples   - Reason out about the offer for escorted referral or bus fare - How if Asha was 21 years old, would the reaction for an offer to be linked to care be different? Why?   **Note:** Pay particular attention to the role of:   - Self-stigma - Family members’ reaction after finding out that she is using C&T services - Community members’ reaction after finding out that she is using C&T services?   What do you think would help to increase the numbers of girls/women who test HIV positive to accept linkage to C&T early enough? Why do you think so?  Probe for:   - *Use of counsellors? Case managers? PEs? HBCs? Self-referral?* - *Enrolment on weekdays/ weekends, Flexible hours?* |  |
| **Acceptability of PrEP** |  | Before we end this discussion, I will like to understand your opinion regarding access to pills which reduce the risk of HIV infection for someone who is HIV negative if taken every day (PrEP)?  Have you heard about that pill (PrEP)?   - If that pill would be available in your community how would you respond? - What would be reasons for people to accept or refuse to use it / [reasons to seek such services?] - Whom would you recommend to provide such pills to adolescent girls/women in your community? Why do you think so? - If a girl/woman who is using this medicine is given extra pills to share with other people in need of it whom do you think she will give? (by priority], why? - What if a person with multiple partners is given the pills to provide to his/her partners who will s/he give to and why? [by priority] - If instead of daily pills PrEP, an injection is provided every two months, would this make a difference? how and why? - How do you think the availability of the pill to prevent against HIV infection may influence sexual behavior? |  |
| **Closing** |  | Is there anything else that we didn’t discuss that you think is important to know about when considering CTC linkage? |  |

End of discussion. Thank participants.

**2.2. Focus Group Discussion Guide: Service providers (Biomed & Nurses)**

**Understanding barriers and facilitators to partner notification, enrolment to C&T and HIV + yield in the Sauti program implementation regions in Tanzania**

Participants’ demographics –Facilitator will fill out the following chart by speaking with each participant prior to initiating the focus group discussion

| **Number** | **Gender** | **Age** | **Level of education** | **Role in Sauti** | **Remarks** |
| --- | --- | --- | --- | --- | --- |
| 1 |  |  |  |  |  |
| 2 |  |  |  |  |  |
| 3 |  |  |  |  |  |
| 4 |  |  |  |  |  |
| 5 |  |  |  |  |  |
| 6 |  |  |  |  |  |
| 7 |  |  |  |  |  |
| 8 |  |  |  |  |  |
| 9 |  |  |  |  |  |
| 10 |  |  |  |  |  |

**Instruction for the facilitators:** These questions are guides for the discussion. In general, please try to follow the question flow outlined below. Participants may take the conversation in a different direction. This is fine, but please ensure that by the end of the session you have covered all of the listed domains.

**Introductory text for group:** Thank you all for coming today and agreeing to speak with us. Everything we discuss today will be confidential so we ask you not to discuss what we talk about with anyone outside of this room. You have kindly agreed for us to make an audio recording of our discussion and in order to protect your privacy, we ask you not to mention your name or anyone else’s name during the session. Instead please use the number/letter that we just assigned to identify yourself when you want to speak.

| **Theme** | **Q** | **Questions** | **Summary** |
| --- | --- | --- | --- |
| **Understanding partner notification** |  | Let’s start by discussing issues related to partner notification in your context  What does partner notification mean to you?  How is this concept relevant in what you do for Sauti?  What are the efforts done by Sauti to enable you to provide appropriate partner notification?  Probe for:   - Do you think you have appropriate skills to perform/facilitate partner notification? Why?   What do you know about IPV tool?   - How do you use it? - Are there challenges related to performing IPV? - What do you think can be done to avert these challenges?   What specific areas would you suggest improvement in the way Sauti perform/facilitate partner notification? Give examples |  |
| **Partner notification** |  | I want to understand a bit more about your experience in performing/facilitating partner notification for your clients.  Think about a typical girl/woman coming to you for the service, and at some point you offer a partner notification. How would she react? Please give some example.  Probe for:   - What do clients say when you offer partner notification - How would be the reaction if the client was tested at the Sauti service delivery point - How would be the reaction if the client was tested at a nearby health facility   What are the challenges you are facing when you perform partner notification activities? |  |
| **Understanding C&T enrolment context** |  | What options do girls/women have for enrolling to care and treatment in this community?   - Probe for preferences in order of importance [use flip chart for participatory ranking of the options)   What are your experiences in providing escorted referral?  What are the reasons that may prevent people from enrolling into care and treatment?  What are the motivations for Sauti key beneficiaries to enrol into care and treatment services?  What would you recommend for Sauti to increase C&T enrollment in this context? |  |
| **Reasons for low yield** |  | Let’s now discuss about the program reach and yield.  Where do Sauti program go for HTC demand creation?  Probe for:   - - Brothels   - Bars, clubs   - Truck stops   - Work places (plantations, mines, industries,   - Time (moonlight)   What are the strategies used in demand creation?  Probe for:   - Peer educators - Home-based care - Key informants etc   In order of importance, please rank the strategies from high yielding to low yielding and their reasons.  What are the challenges for reaching Sauti beneficiaries in your community?  What can be done to reach high number of beneficiaries infected with HIV?  What can be done to increase the number of people who are at higher risk of HIV infection?  What can be done to increase the number of people who are HIV positive to come for HIV testing? |  |
| **Closing** |  | Is there anything else that we didn’t discuss that you think is important to know about when considering CTC linkage? |  |

End of discussion. Thank participants.

**2.3. Focus Group Discussion Guide: Peer educators for AGYW & FSW**

**Understanding barriers and facilitators to partner notification, enrolment to C&T and HIV + yield in the Sauti program implementation regions in Tanzania**

Participants’ demographics –Facilitator will fill out the following chart by speaking with each participant prior to initiating the focus group discussion

| **Number** | **Age** | **Level of education** | **Marital status** | **Main activity/sources of income** | **Remarks** |
| --- | --- | --- | --- | --- | --- |
| 1 |  |  |  |  |  |
| 2 |  |  |  |  |  |
| 3 |  |  |  |  |  |
| 4 |  |  |  |  |  |
| 5 |  |  |  |  |  |
| 6 |  |  |  |  |  |
| 7 |  |  |  |  |  |
| 8 |  |  |  |  |  |
| 9 |  |  |  |  |  |
| 10 |  |  |  |  |  |

**Instruction for the facilitators:** These questions are guides for the discussion. In general, please try to follow the question flow outlined below. Participants may take the conversation in a different direction. This is fine, but please ensure that by the end of the session you have covered all of the listed domains

**Introductory text for group:** Thank you all for coming today and agreeing to speak with us. Everything we discuss today will be confidential so we ask you not to discuss what we talk about with anyone outside of this room. You have kindly agreed for us to make an audio recording of our discussion and in order to protect your privacy, we ask you not to mention your name or anyone else’s name during the session. Instead please use the number/letter that we just assigned to identify yourself when you want to speak.

| **Theme** | **Q** | **Questions** | **Summary** |
| --- | --- | --- | --- |
| **Understanding partner notification** |  | Let us begin by discussing issues related to communication with partners.  How easy or difficult it is for girls/women to discuss issues related to sexual and reproductive health with their sexual partners? Give examples  What do they usually discuss about?  Probe for:   - Protection against pregnancy - HIV testing - Using other means of protection – (to be mentioned)   How do partners react when a girl/woman initiates a discussion about those issues? Give examples.  How if partners would know each other’s HIV status? (*is it something good/bad?)  Probe for:   - What would happen if one is found HIV positive - What would happen if both are HIV positive or negative?   As peer educators, what can you do to help your peers to communicate with their partners about their health status?  Probe for:   - Do you usually talk to them about notifying their partners? Why/why not?   What can be done to ensure that partner notification becomes girls’/women’s behaviour? |  |
| **Context of HIV test & care seeking practice** |  | Since you live and work in this community, I want to understand a bit about girls/women’s decision making process when it comes to test for HIV and use of medicines.  Think about a typical girl/woman in your community, what can influence her to go for a HIV test?  Probe for: [pregnancy, marriage, initiate C&T]  What happens when other people in the community finds out that a girl/woman is using care and treatment services? Explore examples.  What expenses would a girl/woman incur in attending and receiving care and treatment? |  |
| **Care and treatment context** |  | Have you ever heard about care and treatment facilities? [if this was mentioned, re-phrase as:  You mentioned that sometimes girls/women go to care and treatment facilities......   - Are there medicines that one can use for that condition? (Knowledge on availability of ART) - Where can one access care and treatment services in this community? - What are the challenges one can expect in accessing these services? Give examples - What would happen if one delays in seeking care?   What are their experiences in accessing the services there?  Probe for:   - How do they describe the quality of services offered? Give examples - How do they say about healthcare providers there? Pay attention to stigma and discrimination. |  |
| **Barriers and facilitators to C&T enrolment** |  | I'd like to understand the context for enrolling to care and treatment among girls/women.  Some people diagnose HIV positive early, but they do not enroll to C&T. What do you think are the reasons for the delays or not accessing the available services? Give examples  What are the challenges facing girls/women in enrolling to C&T?  Pay particular attention to the role of:   - Self-stigma - What happens when family members find out that she is using C&T services? - What happens when community members find out that she is using C&T services?   What do you think would help to increase the numbers of girls/women who test HIV positive to enroll to C&T? Why do you think so?  Probe for:   - *Use of counsellors? Case managers? PEs? HBCs? Self-referral?* - *Enrollment on weekdays/ weekend, Flexible hours?* |  |
| **Acceptability of community-based services** |  | Have you heard of pills which reduce the risk of HIV infection for someone who is HIV negative if taken every day (PrEP)?     - What would be reasons for people to access or not access such services? - For how many years do you think one could take every day pills to prevent HIV infection? - Whom would you recommend to provide such pills? - What training would this person need? - If an HIV negative MSM who is receiving PrEP for himself would have extra pills to give to another person, to whom would he give them, when and why? - What would you think if an HIV infected person would get PrEP pills to give to his sexual partners, to whom would he give them, when and why? - If instead of daily pills PrEP an injection every two months would be available, in how far would this make a difference?   How do you think PrEP would influence sexual behaviour of MSM? |  |
| **Closing** |  | Is there anything else that we didn’t discuss that you think is important to know in this discussion? |  |

End of discussion. Thank participants.
