# Supplementary material for: Facilitators and barriers to linkage to HIV care and treatment among female sex workers in a community-based HIV prevention intervention in Tanzania: A qualitative study
Source: PLoS One. 2019 Nov 19;14(11):e0219032. doi: 10.1371/journal.pone.0219032 (PMC6863533; doi:10.1371/journal.pone.0219032)
Supplement: S2 Text — (DOC) [file pone.0219032.s002.doc]

**2.1. Muongozo wa majadiliano katka kundi lengwa: FSW vAGYWs**

**Uelewa kuhusu vizuizi na vichocheo vya Utoaji wa Taarifa kwa wenza, Kujisajili ktk Huduma na Matibabu ya VVU na Upatikanaji wa Waathirika wa VVU kwenye Mikoa ambamo Mradi wa Sauti Unatekelezwa, Tanzania**

Taarifa za washiriki – Mlaghabishaji atajaza chati hii kwa kuzungumza na mshiriki mmoja mmoja

| **Namba** | **Umri** | **Kiwango cha elimu** | **Hali ya ndoa** | **Shighuli/vyanzo vya mapato** | **Maoni** |
| --- | --- | --- | --- | --- | --- |
| 1 |  |  |  |  |  |
| 2 |  |  |  |  |  |
| 3 |  |  |  |  |  |
| 4 |  |  |  |  |  |
| 5 |  |  |  |  |  |
| 6 |  |  |  |  |  |
| 7 |  |  |  |  |  |
| 8 |  |  |  |  |  |
| 9 |  |  |  |  |  |
| 10 |  |  |  |  |  |

**Maelezo kwa walaghabishaji:** Maswali haya ni muongozo wa majadiliano. Kwa jumla, tafadhali jaribu kufuata mtiririko wa maswali yaliyoainishwa hapo chini. Washiriki wanaweza kupeleka mjadala kwenda uelekeo tofauti. Hilo ni sawa tu, lakini jaribu kuona kuwa mwisho wa mjadala mada zote zilizoainishwa zimejadiliwa vilivyo.

**Utangulizi:** Tunawashukuruni nyote kwa kuja na kukubali kuzungumza na sisi. Kila tutakachokijadili leo kitakuwa ni siri, kwahiyo tunawaomba kuwa chochote tutakacho kijadili msikijadili na mtu yeyote nje ya chumba hiki. Mmetukubalia kutumia kinasa sauti katika mazungumzo yetu, sasa ili kuhakikisha kuwa kuna usiri, tunawaomba kuto kutaja majina yenu au jina la mtu yeyote wakati wa mjadala. Badala yake tumia namba uliyopewa kujitambulisha wakati wa kuchangia mada.

| **Mada** | **Q** | **Maswali** | **Ufupisho** |
| --- | --- | --- | --- |
| **Uelewa kuhusu Utoaji wa taarifa kwa wenza** |  | Ningependa tuanze kwa kujadili mambo yanayohusu mawasiliano na wapenzi.  Ni mambo gani muhimu ambayo wasichana/wanawake wana jadili na wapenzi wao?  Dadisi kuhusu:   - Kujikinga dhidi ya kupata mimba zisizotarajiwa - Upimaji wa VVU - Matumizi ya njia nyingine za kujikinga – (wataje)   Je, wapenzi wanalichukuliaje endapo msichana/mwanamke anaanzisha mjadala kuhusu mambo hayo? Toa mifano.  Vipi kama wapenzi wangefahamu hali ya maambukizi ya VVU kwa kila mmoja? (*je ni jambo zuri/baya?)  Dadisi kuhusu:   - Nini kinaweza kutokea endapo mmoja ana maambukizi ya VVU - Nini kinaweza kutokea endapo wote wana maambuki au hawana maambukizi?   Ni njia zipi zinaweza kutumika kumfikia mpenzi wako ili kupima VVU? Kwanini?  Dadisi kuhusu:   - Kwa madaktari/manesi kuwasiliana naye/kuwafikia bila kuwajulisha kuhusu hali ya maambukizi ya wapenzi wao? Kwanini mnafikiri hivyo? - Kumleta mpenzi moja kwa moja kupimwa? Kwanini? - Je, kuna msaada wowote unaohitajika ili mpenzi aweze kuja? Kwanini? |  |
| **Upimaji wa VVU na utafutaji wa huduma** |  | Ningependa kuelewa zaidi kuhusu uzoefu wenu katika kufanya maamuzi ya kupima VVU na matumizi ya dawa za kufubaza makali ya VVU katika jamii hii.  Fikiri kuhusu msichana/mwanamke katika jamii, ni mambo gani yanaweza kufanya aende kupima VVU?  Dadisi kuhusu:   - Atajisikiaje endapo atagundulika kuwa ana maambukizi ya VVU? Toa mfano tafadhali - Anaweza kufanya nini mara baada ya kugundua kuwa ana maambukizi ya VVU?   Je, mtu anaweza kufanya nini ili kuendelea kuishi kama kawaida baada ya kugundua kuwa ana maambukizi ya VVU?  Dadisi:   - Je, kuna dawa ambazo mtu anaweza kuzitumia kwa ajili ya hali hiyo? (uelewa kuhusu uwepo wa ART) - Je, mtu anaweza kupata wapi huduma na matibabu ya VVU? - Ni changamoto zipi mtu anaweza kuzitarajia anapo tafuta huduma hizi? Toa mifano - Nini kinaweza kutokea endapo mtu atachelewa kupata huduma hizi? |  |
| **Mazingira ya huduma na matibabu ya VVU** |  | Je, mmewahi kusikia kuhusu vituo vya huduma na matibabu ya VVU? [endapo iligusiwa, badili na kuuliza:  mmetaja kuwa kuna wakati wasichana/wanawake wanakwenda kwenye vituo vya huduma na matibabu ya VVU......  Je, wanakutana na mambo gani wanapotafuta huduma pale?  Dadisi:   - Wanaelezeaje ubora wa huduma zinazotolewa? Toa mifano - Wanawazungumziaje watoa huduma pale? Zingatia kuhusu unyanyapaa na kutengwa.   Ni gharama zipi za kifedha au zisizo za kifedha msichana/mwanamke anaingia endapo ataamua kuhudhulia na kutafuta huduma na matibabu ya VVU? |  |
| **Vikwazo na vichocheo vya kujiunga na huduma endelevu kwa watu waliogundulika kuwa na maambukizi ya VVU** |  | Ningependa kuelewa mazingira ya kujiunga na huduma za matibabu ya VVU kwa wasichana/wanawake. Hebu tufikiri kuhusu msichana anayeitwa Asha anayeishi hapa kijijini/mjini.  **Soma kielelezo kwa nguvu:**  ***“Asha ni msichana mwenye miaka 17. Anaishi na mwanaume kinyumba na hana chanzo chochote cha mapato zaidi ya pesa anazopata kutoka kwa mumewe. Asha alikubali kupimwa VVU kupitia mradi wa Sauti, na akagundulika kuwa na maambukizi. Baada ya vipimo, mtoa huduma wa mradi alimwambia kuwa wangempeleka kwenye kituo cha afya cha karibu palepale kijijini/mjini kwa ajili ya ushauri nasaha na kuanzishiwa matibabu. Hata hivyo, Asha hakukubali msaada huo. Asha alipewa kiasi cha Shilingi 5,000/- ili aende mwenyewe kituo cha afya kwa ajili ya huduma. Baada ya siku sita wahudumu wa mradi walikwenda kumtembelea Asha ili kujua maendeleo yake, lakini waligundua kuwa Asha hakwenda kituo cha afya kwa ajili ya huduma ya ushauri nasaha na matibabu”.***  Nini kinaweza kuwa kilimzuia Asha kwenda kituo cha afya ili kuanzishiwa huduma na matibabu? Toa mifano   - Dadisi kuhusu kupelekwa kituo cha afya na wahudumu wa mradi au kupewa nauli - Vipi kama Asha angekuwa na umri wa miaka 21, je mwitikio kuhusu kupelekwa kituo cha afya na wahudumu wa mradi ungekuwa tofauti? Kwanini?   **Zingatia:** Fuatilia kwa karibu kuhusu:   - Kujinyanyapaa - Jinsi wanafamilia watakavyomuona endapo wakigundua anatumia huduma na matibabu ya VVU - Jinsi jamii itakavyomuona endapo wakigundua anatumia huduma na matibabu ya VVU   Mnafikiri nini kinaweza kufanyika ili kuongeza idadi ya wasichana/wanawake wanaogundulika kuwa na maambukizi ya VVU kuanza kujiunga na huduma VVU mapema? Kwanini unafikiri hivyo?  Dadisi kuhusu:   - *Kutumia washauri nasaha? Watoa huduma majumbani? Wauelisha rika? Kuenda wenyewe?* - *Kujiunga siku za katikati ya wiki/siku za mwisho wa wiki, masaa yoyote/wakati wowote?* |  |
| **Kukubalika kwa matumizi ya PrEP** |  | Kumalizia majadiliano yetu, ningependa tujadili kidogo kuhusu dawa inayoweza kutumika kupunguza uwezekano wa kupata maambukizi ya VVU  Je, mmewahi kusikia kuhusu dawa hiyo?   - Endapo dawa hiyo itapatikana kwenye jamii yenu, mngeipokeaje? - Je, mnadhani ni sababu zipi zingewafanya watu waitumie au kuto kuitumia dawa hiyo? - Je, mngependa nani ahusike katika kutoa dawa hiyo kwa wasichana/wanawake katika jamii hii? Kwanini mnafikiri hivyo? Itolewe wapi? - Endapo msichana/mwanamke anayetumia dawa hizi angepewa dawa za ziada ili awapatie watu wengine wenye mahitaji hayo, je mnadhani angempatia nani? Kwanini? - Vipi endapo mtu aliyeambukizwa na ana wapenzi wengi akapata vidonge vya PrEP ili kuwapa wapenzi wake, je atampa nani? Na kwanini? - Je, endapo dawa ya kinga ikatolewa kwa njia ya sindano kila baada ya miezi miwili, watu wangeipokeaje ukilinganisha na kidonge kinachotumika kila siku? Kwanini? - Je, mnadhani ni kwa namna gani uwepo wa dawa za kinga dhidi ya maambukizi ya VVU kunawezaje kuathiri mahusiano ya kinongo miongoni mwa watu. |  |
| **Hitimisho** |  | Je, kuna jambo lolote ambalo hatujalijadili ambalo mnadhani ni muhimu kwetu kujua wakati mtu anafikiri kuanza huduma na matibabu ya VVU? |  |

Mwisho wa mjadala. Washukuru washiriki.

**2.2. Muongozo wa majadiliano katika kundi lengwa: Watoa huduma (Biomed & Nurses)**

**Uelewa kuhusu vizuizi na vichocheo vya Utoaji wa Taarifa kwa wenza, Kujisajili ktk Huduma na Matibabu ya VVU na Upatikanaji wa Waathirika wa VVU kwenye Mikoa ambamo Mradi wa Sauti Unatekelezwa, Tanzania**

Taarifa za washiriki – Mlaghabishaji atajaza chati hii kwa kuzungumza na mshiriki mmoja mmoja

| **Namba** | **Jinsia** | **Umri** | **Kiwango cha elimu** | **Majukumu ndani ya Sauti** | **Maoni** |
| --- | --- | --- | --- | --- | --- |
| 1 |  |  |  |  |  |
| 2 |  |  |  |  |  |
| 3 |  |  |  |  |  |
| 4 |  |  |  |  |  |
| 5 |  |  |  |  |  |
| 6 |  |  |  |  |  |
| 7 |  |  |  |  |  |
| 8 |  |  |  |  |  |
| 9 |  |  |  |  |  |
| 10 |  |  |  |  |  |

**Maelezo kwa walaghabishaji:** Maswali haya ni muongozo wa majadiliano. Kwa jumla, tafadhali jaribu kufuata mtiririko wa maswali yaliyoainishwa hapo chini. Washiriki wanaweza kupeleka mjadala kwenda uelekeo tofauti. Hilo ni sawa tu, lakini jaribu kuona kuwa mwisho wa mjadala mad azote zilizoainishwa zimejadiliwa vilivyo.

**Utangulizi:** Tunawashukuruni nyote kwa kuja na kukubali kuzungumza na sisi. Kila tutakachokijadili leo kitakuwa ni siri, kwahiyo tunawaomba kuwa chochote tutakacho kijadili msikijadili na mtu yeyote nje ya chumba hiki. Mmetukubalia kutumia kinasa sauti katika mazungumzo yetu, sasa ili kuhakikisha kuwa kuna usiri, tunawaomba kuto kutaja majina yenu au jina la mtu yeyote wakati wa mjadala. Badala yake tumia namba uliyopewa kujitambulisha wakati wa kuchangia mada.

| **Mada** | **Q** | **Maswali** | **Ufupisho** |
| --- | --- | --- | --- |
| **Uelewa kuhusu Utoaji wa taarifa kwa wenza** |  | Ningependa tuanze kwa kujadili mambo yanayohusu mawasiliano kwa wapenzi katika mazingira yenu.  Je, mnaelewa nini kuhusu kutoa taarifa kwa mpenzi (partner notification)?  Kwa vipi swala hili linawahusu katika mazingira yenu ya kazi kwenye mradi wa Sauti?  Ni jitihada gani mbazo Sauti imezifanya ili kuwawezesha muweze kufanya /kuwawezesha wateja wenu kutoa taarifa kwa wapenzi wao?  Dadisi:   - Je, mnadhani mna ujuzi wa kutosha ili kutekeleza jukumu hilo au kuwawezesha wateja wenu kutoa taarifa kwa wapenzi wao? Kwanini?   Mnaelewa nini kuhusu muongozo wa unaotumika kupima vitendo vya ukatili miongoni mwa wapenzi (IPV tool)?   - Mnautumiaje mwongozo huo? - Je, kuna changamoto zozote mnazozipata kuutumia (IPV tool)? - Mnadhani nini kinaweza kufanyika ili kupunguza changamoto hizo?   Ni maeneo gani hasa ambayo mngependekeza yaboreshwe kuhusiana na namna ya utoaji wa taarifa kwa wateja wenu? Toa mifano |  |
|  |  | Napenda kuelewa zaidi kuhusu uzoefu wenu katika kufanya/kuwezesha utoaji wa taarifa kwa wenzi wa wateja wenu.  Fikiri kuhusu msichana/mwanamke wa kawaida anayekuja kupata huduma, na wakati fulani unataka kutoa taarifa kwa mwenzi wake. Je, anaweza kulichukuliaje? Tafadhali toa baadhi ya mifano.  Dadisi kuhusu:   - Je, wateja wenu wanasemaje mnapotaka taarifa itolewe kwa wapenzi wao? - Anaweza kufanyaje endapo atakuwa amepimwa katika kituo cha karibu cha Sauti cha kutolea huduma? - Anaweza kufanyaje endapo atakuwa amepimwa katika kituo cha karibu cha CTC?   Ni changamoto gani mnazopata manapofanya shughuli ya utoaji wa taarifa kwa mwezi wa mteja wenu? |  |
| **Uelewa kuhusu kujiunga na huduma ya VVU** |  | Ni sehemu zipi ambazo wasichana/wanawake wanaweza kuanzishiwa huduma na matibabu ya VVU katika jamii hii?   - Dadisi – ni wapi panapopendelewa hasa? [tumia flip chart kuorodhesha kwa umuhimu]   Je, mnaonaje (uzoefu wenu) katika kutoa huduma za kusindikiza wateja kwenda kwenye vituo vya huduma na matibabu?  Ni sababu gani zinazoweza kuzuia watu kujiunga katika huduma na matibabu ya VVU?  Ni mambo gani yanayowasukuma walengwa wa mradi wa Sauti kujiunga na huduma na matibabu ya VVU?  Je, nini mapendekezo yenu kwa mradi wa Sauti ili kuongeza idadi ya walengwa wa mradi wanaojiunga na kutumia huduma na matibabu kwenye jamii hii? |  |
| **Sababu ya uibuaji mdogo wa wanaoishi na maambukizi ya UKIMWI**  **[Reasons for low yield]** |  | Sasa tujadili jinsi mradi unavyowafikia wateja na matokeo yake.  Je, mradi wa Sauti unakwenda wapi kuhamasisha kuhusu ushauri nasaha na upimaji wa VVU?  Dadisi:   - - Madangulo   - Baa, Klub   - Vituo vya magari ya mizigo   - Mahali pa kazi (mashamba makubwa, migodi ……..)   - Wakati (usiku ….)   Ni mbinu zipi zinatumika kuhamasisha kuhusu ushauri nasaha na upimaji wa VVU?  Dadisi:   - Waelimishaji rika - Watoa huduma majumba - Watoa taarifa mahsusi   Kwa mtiriko wa umuhimu, tafadhali orodhesha mbinu hizo kuanzia zinazoleta matokeo makubwa na sababu zake.  Kuna changamoto zipi katika kuwafikia walengwa wa mradi wa Sauti katika jamii hii?  Nini kinaweza kufanyika ili kuibua idadi kubwa ya walengwa wenye maambukizi ya VVU?  Je, mradi wa Sauti unaweza kufanya nini ili kuwafikia wasichana wanaojiuza ambao hawawezi kupatikana kwenye madangulo, mitaani n.k., |  |
| **Hitimisho** |  | Je, kuna jambo lolote ambalo hatujalijadili ambalo mnadhani ni muhimu kwetu kujua wakati mtu anafikiri kuanza huduma na matibabu ya VVU? |  |

Mwisho wa mjadala. Washukuru washiriki.

**2.3. Mwongozo wa majadiliano shirikishi ya kikundi: Watoa huduma ktk Jamii**

**Uelewa kuhusu vizuizi na vichocheo vya Utoaji wa Taarifa kwa wenza, Kujisajili ktk Huduma na Matibabu ya VVU na Upatikanaji wa Waathirika wa VVU kwenye Mikoa ambamo Mradi wa Sauti Unatekelezwa, Tanzania**

Taarifa binafsi za washiriki –mwezeshaji wa kikundi atajaza fomu ifuatayo kwa kuzungumza na kila mshiriki kabla ya kuanza kwa mahojiano shirikishi ya kikundi

| **Namba** | **Jinsi** | **Umri** | **Kiwango cha elimu** | **Hali ya ndo** | **Maoni** |
| --- | --- | --- | --- | --- | --- |
| 1 |  |  |  |  |  |
| 2 |  |  |  |  |  |
| 3 |  |  |  |  |  |
| 4 |  |  |  |  |  |
| 5 |  |  |  |  |  |
| 6 |  |  |  |  |  |
| 7 |  |  |  |  |  |
| 8 |  |  |  |  |  |
| 9 |  |  |  |  |  |
| 10 |  |  |  |  |  |

**Utangulizi wa mwezeshaji:** Maswali yafuatayo ni kwa ajili ya kuongoza majadiliano ya kikundi. Zingatia kufuata mtiririko wa maswali yafuatayo hapo chini. Wakati mwigine washiriki wanaweza kuyapeleka mazungumzo nje ya mtiririko huu. Hilo ni jabo linalokubaliki, lakini tafadhali hakikisha kuwa mwishoni mwa mahojiano maswali hoja zote ziwe ziimezungumzwa/ maswali yote yawe yamejibiwa.

**Taarifa za awali za kutambulisha mjadala la kikundi:** Tunawashukuru wote kwa kukubali kwenu kufika kwa ajili ya mazungumzo haya na sisi leo. Mambo yote tutakayozungumza leo yatachukuliwa kuwa siri na kwa hiyo tunawaomba msiende kuyazungumza na mtu yeyoye kuhusu mambo haya yatakayokuwa yamesemwa kwenye majadiliano ya kikundi. Ili kutotambulisha nani amesema nini kwenye majadiliano yetu tunawaomba msitajane majina halisi kwenye majadiliano haya wala msitaje jina halisia la mtu yeyote kwenye majadiliano haya. Badala ya kutaja jina la mtu, unaweza kutumia namba kama utambulisho wa mtu, au herufi ambazo tutakuwa tumewapa kwa ajili ya majadiliano haya.

| **Mada** | **Q** | **Maswali** | **muhtasari** |
| --- | --- | --- | --- |
| **Uelewa kuhusu Utoaji wa taarifa kwa wenza** |  | Tuanze kwa kujadili kuhusu hili suala la mawasiliano iongoni mwa wapenzi/wenza.  Kuna urahisi au ugumu gani kwa msichana /mwanamke kujadili masuala yanayohusiana mahusiano ya kimapenzi na afya ya uzazi na wenza wao? Toa mifano halisi.  Ni mambo gani muhimu ambayo wasichana/wanawake wana jadili na wapenzi wao?  Dadidi kuhusu:   - Kujikinga dhidi ya kupata mimba zisizotarajiwa - Upimaji wa VVU - Matumizi ya njia nyingine za kujikinga – (wataje)   Je, wapenzi wanalichukuliaje endapo msichana/mwanamke anaanzisha mjadala kuhusu mambo hayo? Toa mifano.  Vipi kama wapenzi wakifahamu hali ya maambukizi ya VVU kwa kila mmoja? (*je ni jambo zuri/baya?)  Dadisi kuhusu:   - Nini kinaweza kutokea endapo mmoja ana maambukizi ya VVU - Nini kinaweza kutokea endapo wote wana maambuki au hawana maambukizi?   Kama waelimisha rika, mnaweza kufanya nini ili kuwasaidia wana rika kuwasiliana na wenzi wao kuusu hali yao ya maambukizi ya VVU?   - Je, kwa kawaida huwa mnaongea na wanarika wenzenu juu ya kuwafahamisha wenzi wao kuhusu hali yao ya maambukizi ya VVU? Eleza kwa nini unafanya hivyo/hufanyi hivyo   Ni nini kifanyike ili kuhakikisha kuwa wasichana/wanawake wanakuwa na tabia za kuwataarifu wenza wao kuhusu hali/hadhi yao ya maambukizi ya VVU |  |
| **Upimaji wa VVU na utafutaji wa huduma** |  | Kwa kuwa mnaishi na kufanya kazi katika jamii hii, ningependa kujua zaidi kuhusu ushiriki wa wasichana/wanawake katika kufanya maamuzi inapokuja suala la upimaji VVU na matumizi ya madawa.  Fikiri kuhusu msichana/mwanamke katika jamii, ni mambo gani yanaweza kufanya aende kupima VVU?  Dadisi kuhusu: - ujauzito, ndoa, kuanza huduma na matibabu ya VVU  Nini kinatokea endapo watu wengine katika jamii wakigundua kuwa msichana/mwanamke anatumia huduma na matibabu ya VVU? Toa mifano  Ni gharama zipi za kifedha au zisizo za kifedha msichana/mwanamke anaingia endapo ataamua kuhudhuria na kutafuta huduma na matibabu ya VVU? |  |
| **Mazingira ya huduma na matibabu ya VVU** |  | Je, mmewahi kusikia kuhusu vituo vya huduma na matibabu ya VVU? [endapo iligusiwa, badili na kuuliza:  mmetaja kuwa kuna wakati wasichana/wanawake wanakwenda kwenye vituo vya huduma na matibabu ya VVU......   - Je, kuna dawa ambazo mtu anaweza kuzitumia kwa ajili ya hali hiyo? (uelewa kuhusu uwepo wa ART) - Je, mtu anaweza kupata wapi huduma na matibabu ya VVU? - Ni changamoto zipi mtu anaweza kuzitarajia anapo tafuta huduma hizi? Toa mifano - Nini kinaweza kutokea endapo mtu atachelewa kupata huduma hizi?   Je, wanakutana na mambo gani wanapotafuta huduma pale?  Dadisi:   - Wanaelezeaje ubora wa huduma zinazotolewa? Toa mifano - Wanawazungumziaje watoa huduma pale? Zingatia kuhusu unyanyapaa na kutengwa. |  |
| **Vikwazo na vichocheo vya kujiunga na huduma endelevu kwa watu wanaoishi na maambukizi ya VVU** |  | Ningependa kuelewa mazingira ya kujiunga na huduma na matibabu ya VVU kwa wasichana/wanawake.  Baadhi ya watu hupima na kugundua kuwa wana maambukizi ya VVU mapema, lakini hawajiungi na huduma na matubabu ya VVU. Mnafikiri kuna sababu gani zinazofanya wachelewe au wasitafute huduma zilizopo? Toa mifano  Ni changamoto gani wanazozipata wasichana/wanawake katika kujiunga na huduma na matibabu ya VVU?  Dadisi na fuatilia zaidi kuhusu:   - Kujinyanyapaa - Nini kinachotokea endapo wanafamilia wakigundua kuwa anatumia huduma na matibabu ya VVU? - Nini kinachotokea endapo watu katika jamii wakigundua kuwa anatumia huduma na matibabu ya VVU? Toa mfano   Mnafikiri nini kinaweza kufanyika ili kuongeza idadi ya wasichana/wanawake wanaogundulika kuwa na maambukizi ya VVU kuanza kujiunga na huduma VVU mapema? Kwanini unafikiri hivyo?  Dadisi kuhusu:   - *Kutumia washauri nasaha? Watoa huduma majumbani? Wauelisha rika? Kuenda wenyewe?* - *Kujiunga siku za katikati ya wiki/siku za mwisho wa wiki, masaa yoyote/wakati wowote?* |  |
| **Kukubalika kwa matumizi ya PrEP** |  | Kumalizia majadiliano yetu, ningependa tujadili kidogo kuhusu dawa inayoweza kutumika kupunguza uwezekano wa kupata maambukizi ya VVU  Je, mmewahi kusikia kuhusu dawa hiyo?   - Endapo dawa hiyo itapatikana kwenye jamii yenu, mngeipokeaje? - Je, mnadhani ni sababu zipi zingewafanya watu waitumie au kuto kuitumia dawa hiyo? - Je, mngependa nani ahusike katika kutoa dawa hiyo kwa wasichana/wanawake katika jamii hii? Kwanini mnafikiri hivyo? Itolewe wapi? - Endapo msichana/mwanamke anayetumia dawa hizi angepewa dawa za ziada ili awapatie watu wengine wenye mahitaji hayo, je mnadhani angempatia nani? Kwanini? - Vipi endapo mtu aliyeambukizwa na ana wapenzi wengi akapata vidonge vya PrEP ili kuwapa wapenzi wake, je atampa nani? Na kwanini? - Je, endapo dawa ya kinga ikatolewa kwa njia ya sindano kila baada ya miezi miwili, watu wangeipokeaje ukilinganisha na kidonge kinachotumika kila siku? Kwanini?   Je, mnadhani ni kwa namna gani uwepo wa dawa za kinga dhidi ya maambukizi ya VVU kunawezaje kuathiri mahusiano ya kinongo miongoni mwa watu. |  |
| **Hitimisho** |  | Je, kuna jambo lolote ambalo hatujalijadili ambalo mnadhani ni muhimu kwetu kujua wakati mtu anafikiri kuanza huduma na matibabu ya VVU? |  |

Mwisho wa mjadala. Washukuru washiriki.
