# Supplementary material for: Facilitators and barriers to linkage to HIV care and treatment among female sex workers in a community-based HIV prevention intervention in Tanzania: A qualitative study
Source: PLoS One. 2019 Nov 19;14(11):e0219032. doi: 10.1371/journal.pone.0219032 (PMC6863533; doi:10.1371/journal.pone.0219032)
Supplement: S3 Text — (DOCX) [file pone.0219032.s003.docx]

# S1- PGD coding scheme summary

## Facilitators

- FSW-friendly providers
- Stories on presence of respectful providers motivates
- Knowledgeable about the needs of FSWs
- Less stigmatizing
- Approachable
- Confidential
- Provision of transport
- Addressed issues of financial constraints
- Motivated to link as soon as diagnosed HIV +ve
- Peer educators
- Provide escorted referral
- Source of HIC care and treatment information
- Knowledgeable of who to contact at health facility
- Peer support networks
- Encourage
- Feeling of less alone
- Feeling less stigmatised

## Barriers

- **System factors**
- Confidentiality issues
- Providers telling other people about FSWs HIV status
- Providers alerting friends not having sexual relationship with FSW testing HIV positive
- Peers and HBCs disclosing FSWs HIV status leading to stigmatisation
- Long pre-enrolment period
- Three days’ pre-enrolment alerted other about their HIV status
- Some absconded even before they initiate treatment
- Unintegrated service delivery
- Having a separate room/block for HIV care is a cause for stigma
- **Societal**
- Myths and rumours about ARV
- Caused horrifying dreams
- Lack of appetite
- Caused sadden death
- Stigma associated with receiving HIV care
- Derogatory naming
- discrimination
- **Individual factors**
- Perceived health status
- Not feeling ill
- Spoiling relationships with partners/clients
